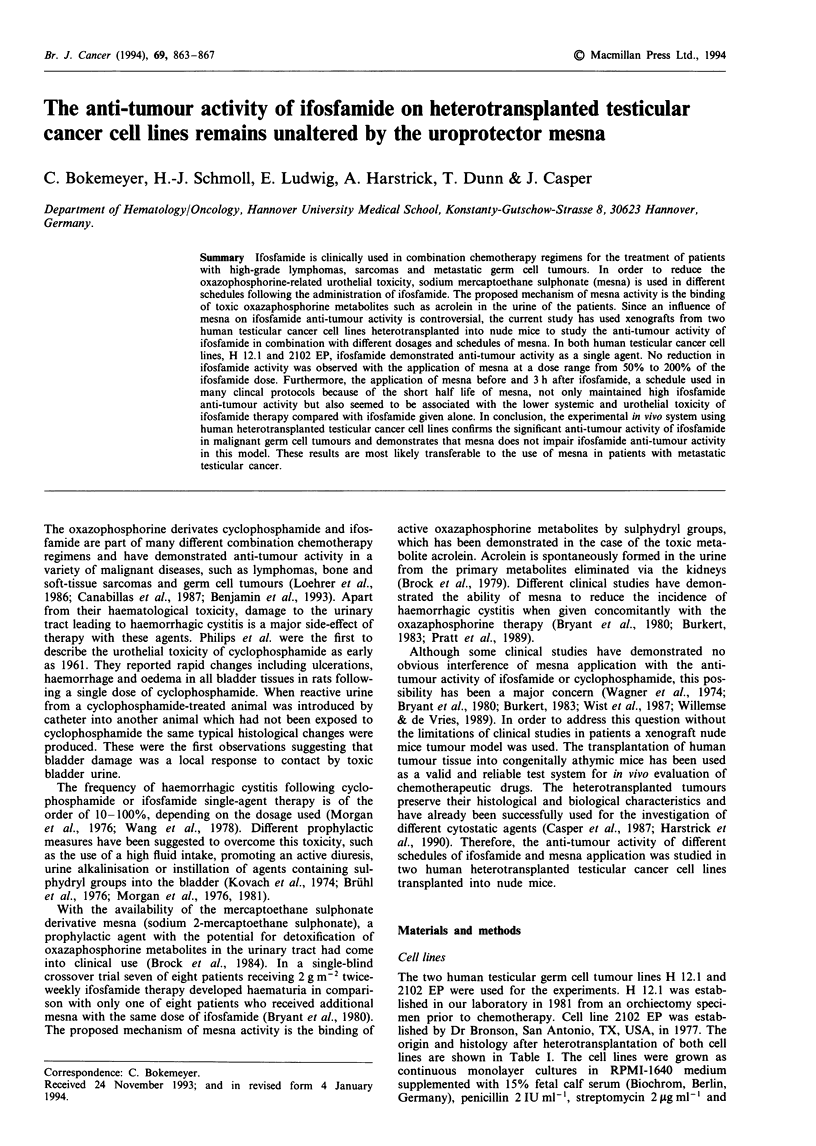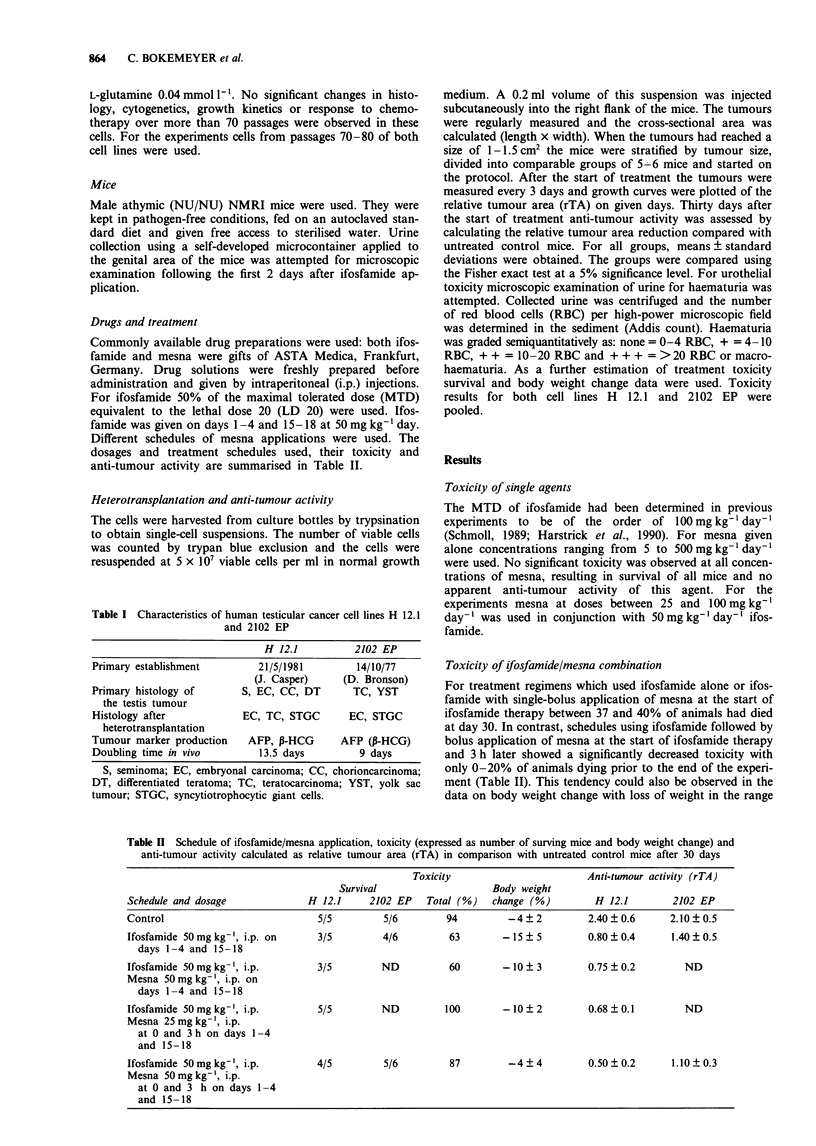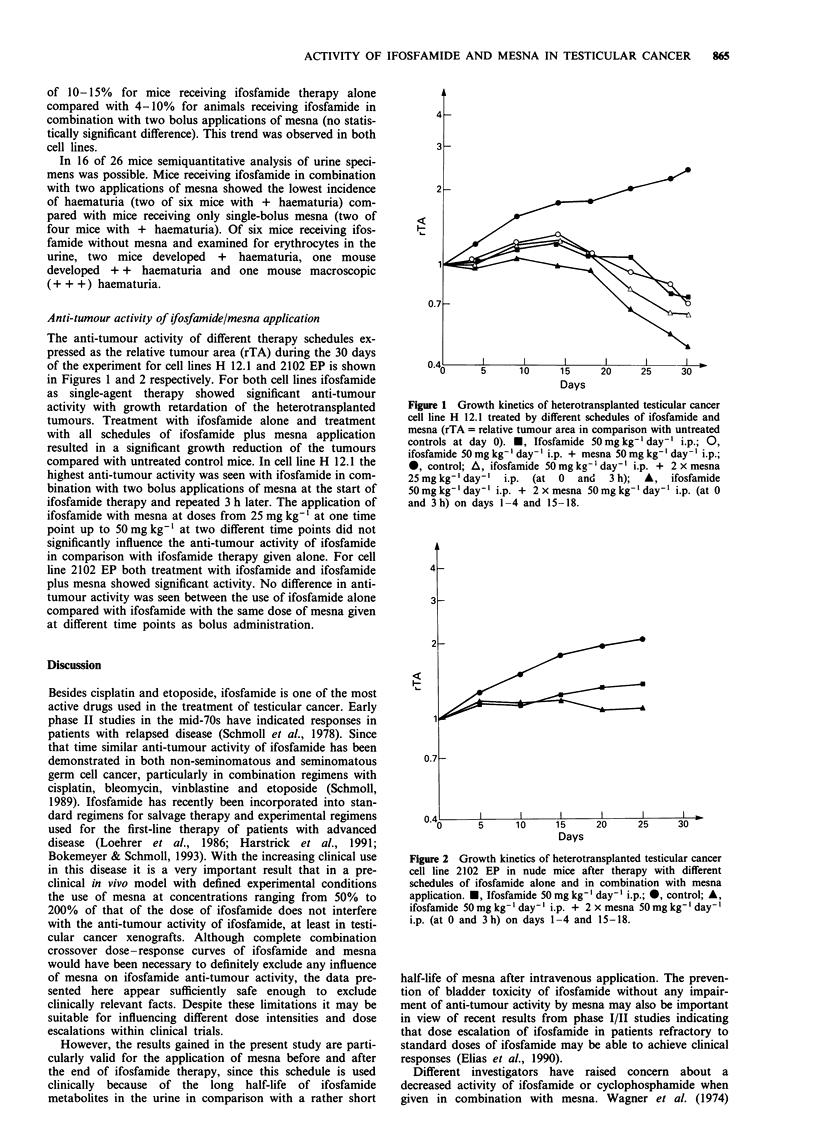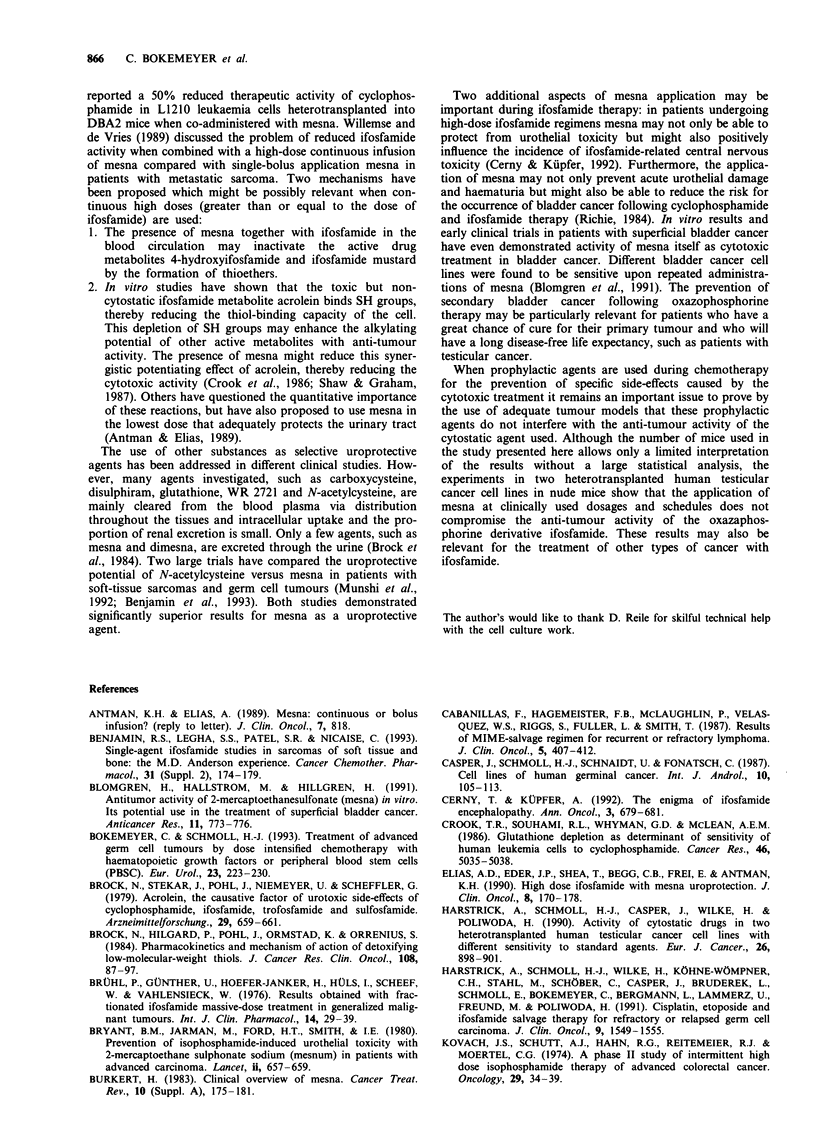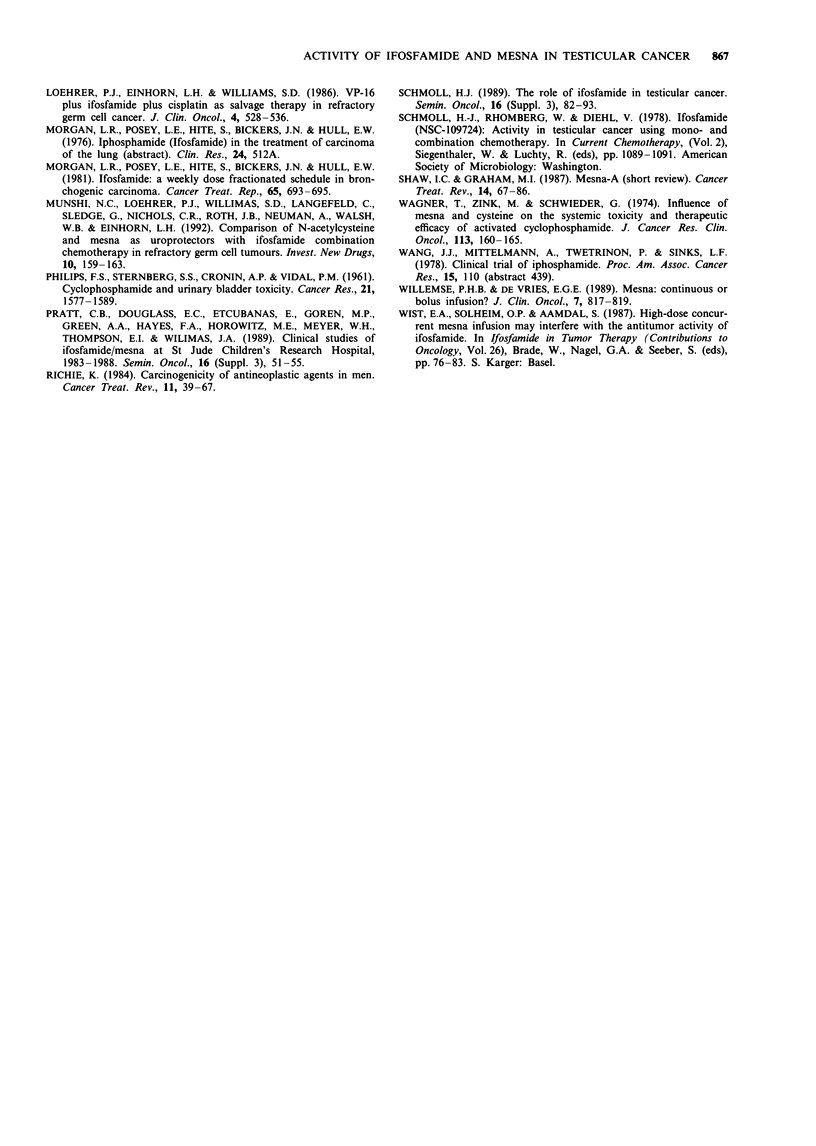# The anti-tumour activity of ifosfamide on heterotransplanted testicular cancer cell lines remains unaltered by the uroprotector mesna.

**DOI:** 10.1038/bjc.1994.167

**Published:** 1994-05

**Authors:** C. Bokemeyer, H. J. Schmoll, E. Ludwig, A. Harstrick, T. Dunn, J. Casper

**Affiliations:** Department of Hematology/Oncology, Hannover University Medical School, Germany.

## Abstract

Ifosfamide is clinically used in combination chemotherapy regimens for the treatment of patients with high-grade lymphomas, sarcomas and metastatic germ cell tumours. In order to reduce the oxazophosphorine-related urothelial toxicity, sodium mercaptoethane sulphonate (mesna) is used in different schedules following the administration of ifosfamide. The proposed mechanism of mesna activity is the binding of toxic oxazaphosphorine metabolites such as acrolein in the urine of the patients. Since an influence of mesna on ifosfamide anti-tumour activity is controversial, the current study has used xenografts from two human testicular cancer cell lines heterotransplanted into nude mice to study the anti-tumour activity of ifosfamide in combination with different dosages and schedules of mesna. In both human testicular cancer cell lines, H 12.1 and 2102 EP, ifosfamide demonstrated anti-tumour activity as a single agent. No reduction in ifosfamide activity was observed with the application of mesna at a dose range from 50% to 200% of the ifosfamide dose. Furthermore, the application of mesna before and 3 h after ifosfamide, a schedule used in many clinical protocols because of the short half life of mesna, not only maintained high ifosfamide anti-tumour activity but also seemed to be associated with the lower systemic and urothelial toxicity of ifosfamide therapy compared with ifosfamide given alone. In conclusion, the experimental in vivo system using human heterotransplanted testicular cancer cell lines confirms the significant anti-tumour activity of ifosfamide in malignant germ cell tumours and demonstrates that mesna does not impair ifosfamide anti-tumour activity in this model. These results are most likely transferable to the use of mesna in patients with metastatic testicular cancer.


Br. J. Cancer (1994), 69, 863–867 © Macmillan Press Ltd., 1994

### The anti-tumour activity of ifosfamide on heterotransplanted testicular cancer cell lines remains unaltered by the uroprotector mesna

C. Bokemeyer, H.-J. Schmoll, E. Ludwig, A. Harstrick, T. Dunn & J. Casper

*Department of Hematology/Oncology, Hannover University Medical School, Konstanty-Gutschow-Strasse 8, 30623 Hannover, Germany.*

**Summary** Ifosfamide is clinically used in combination chemotherapy regimens for the treatment of patients with high-grade lymphomas, sarcomas and metastatic germ cell tumours. In order to reduce the oxazophosphorine-related urothelial toxicity, sodium mercaptoethane sulphonate (mesna) is used in different schedules following the administration of ifosfamide. The proposed mechanism of mesna activity is the binding of toxic oxazaphosphorine metabolites such as acrolein in the urine of the patients. Since an influence of mesna on ifosfamide anti-tumour activity is controversial, the current study has used xenografts from two human testicular cancer cell lines heterotransplanted into nude mice to study the anti-tumour activity of ifosfamide in combination with different dosages and schedules of mesna. In both human testicular cancer cell lines, H 12.1 and 2102 EP, ifosfamide demonstrated anti-tumour activity as a single agent. No reduction in ifosfamide activity was observed with the application of mesna at a dose range from 50% to 200% of the ifosfamide dose. Furthermore, the application of mesna before and 3 h after ifosfamide, a schedule used in many clincal protocols because of the short half life of mesna, not only maintained high ifosfamide anti-tumour activity but also seemed to be associated with the lower systemic and urothelial toxicity of ifosfamide therapy compared with ifosfamide given alone. In conclusion, the experimental *in vivo* system using human heterotransplanted testicular cancer cell lines confirms the significant anti-tumour activity of ifosfamide in malignant germ cell tumours and demonstrates that mesna does not impair ifosfamide anti-tumour activity in this model. These results are most likely transferable to the use of mesna in patients with metastatic testicular cancer.

The oxazophosphorine derivates cyclophosphamide and ifosfamide are part of many different combination chemotherapy regimens and have demonstrated anti-tumour activity in a variety of malignant diseases, such as lymphomas, bone and soft-tissue sarcomas and germ cell tumours (Loehrer *et al.*, 1986; Canabillas *et al.*, 1987; Benjamin *et al.*, 1993). Apart from their haematological toxicity, damage to the urinary tract leading to haemorrhagic cystitis is a major side-effect of therapy with these agents. Philips *et al.* were the first to describe the urothelial toxicity of cyclophosphamide as early as 1961. They reported rapid changes including ulcerations, haemorrhage and oedema in all bladder tissues in rats following a single dose of cyclophosphamide. When reactive urine from a cyclophosphamide-treated animal was introduced by catheter into another animal which had not been exposed to cyclophosphamide the same typical histological changes were produced. These were the first observations suggesting that bladder damage was a local response to contact by toxic bladder urine.

The frequency of haemorrhagic cystitis following cyclophosphamide or ifosfamide single-agent therapy is of the order of 10–100%, depending on the dosage used (Morgan *et al.*, 1976; Wang *et al.*, 1978). Different prophylactic measures have been suggested to overcome this toxicity, such as the use of a high fluid intake, promoting an active diuresis, urine alkalinisation or instillation of agents containing sulphydryl groups into the bladder (Kovach *et al.*, 1974; Brühl *et al.*, 1976; Morgan *et al.*, 1976, 1981).

With the availability of the mercaptoethane sulphonate derivative mesna (sodium 2-mercaptoethane sulphonate), a prophylactic agent with the potential for detoxification of oxazaphosphorine metabolites in the urinary tract had come into clinical use (Brock *et al.*, 1984). In a single-blind crossover trial seven of eight patients receiving 2 g $m^{-2}$ twice-weekly ifosfamide therapy developed haematuria in comparison with only one of eight patients who received additional mesna with the same dose of ifosfamide (Bryant *et al.*, 1980). The proposed mechanism of mesna activity is the binding of active oxazaphosphorine metabolites by sulphydryl groups, which has been demonstrated in the case of the toxic metabolite acrolein. Acrolein is spontaneously formed in the urine from the primary metabolites eliminated via the kidneys (Brock *et al.*, 1979). Different clinical studies have demonstrated the ability of mesna to reduce the incidence of haemorrhagic cystitis when given concomitantly with the oxazaphosphorine therapy (Bryant *et al.*, 1980; Burkert, 1983; Pratt *et al.*, 1989).

Although some clinical studies have demonstrated no obvious interference of mesna application with the anti-tumour activity of ifosfamide or cyclophosphamide, this possibility has been a major concern (Wagner *et al.*, 1974; Bryant *et al.*, 1980; Burkert, 1983; Wist *et al.*, 1987; Willemse & de Vries, 1989). In order to address this question without the limitations of clinical studies in patients a xenograft nude mice tumour model was used. The transplantation of human tumour tissue into congenitally athymic mice has been used as a valid and reliable test system for *in vivo* evaluation of chemotherapeutic drugs. The heterotransplanted tumours preserve their histological and biological characteristics and have already been successfully used for the investigation of different cytostatic agents (Casper *et al.*, 1987; Harstrick *et al.*, 1990). Therefore, the anti-tumour activity of different schedules of ifosfamide and mesna application was studied in two human heterotransplanted testicular cancer cell lines transplanted into nude mice.

### Materials and methods

### Cell lines

The two human testicular germ cell tumour lines H 12.1 and 2102 EP were used for the experiments. H 12.1 was established in our laboratory in 1981 from an orchiectomy specimen prior to chemotherapy. Cell line 2102 EP was established by Dr Bronson, San Antonio, TX, USA, in 1977. The origin and histology after heterotransplantation of both cell lines are shown in Table I. The cell lines were grown as continuous monolayer cultures in RPMI-1640 medium supplemented with 15% fetal calf serum (Biochrom, Berlin, Germany), penicillin 2 IU $ml^{-1}$, streptomycin 2 $\mu g\ ml^{-1}$ and

Correspondence: C. Bokemeyer.
Received 24 November 1993; and in revised form 4 January 1994.

L-glutamine 0.04 mmol $l^{-1}$. No significant changes in histology, cytogenetics, growth kinetics or response to chemotherapy over more than 70 passages were observed in these cells. For the experiments cells from passages 70–80 of both cell lines were used.

### *Mice*

Male athymic (NU/NU) NMRI mice were used. They were kept in pathogen-free conditions, fed on an autoclaved standard diet and given free access to sterilised water. Urine collection using a self-developed microcontainer applied to the genital area of the mice was attempted for microscopic examination following the first 2 days after ifosfamide application.

### *Drugs and treatment*

Commonly available drug preparations were used: both ifosfamide and mesna were gifts of ASTA Medica, Frankfurt, Germany. Drug solutions were freshly prepared before administration and given by intraperitoneal (i.p.) injections. For ifosfamide 50% of the maximal tolerated dose (MTD) equivalent to the lethal dose 20 (LD 20) were used. Ifosfamide was given on days 1–4 and 15–18 at 50 mg $kg^{-1}$ day. Different schedules of mesna applications were used. The dosages and treatment schedules used, their toxicity and anti-tumour activity are summarised in Table II.

### *Heterotransplantation and anti-tumour activity*

The cells were harvested from culture bottles by trypsination to obtain single-cell suspensions. The number of viable cells was counted by trypan blue exclusion and the cells were resuspended at $5 \times 10^7$ viable cells per ml in normal growth medium. A 0.2 ml volume of this suspension was injected subcutaneously into the right flank of the mice. The tumours were regularly measured and the cross-sectional area was calculated (length × width). When the tumours had reached a size of 1–1.5 $cm^2$ the mice were stratified by tumour size, divided into comparable groups of 5–6 mice and started on the protocol. After the start of treatment the tumours were measured every 3 days and growth curves were plotted of the relative tumour area (rTA) on given days. Thirty days after the start of treatment anti-tumour activity was assessed by calculating the relative tumour area reduction compared with untreated control mice. For all groups, means ± standard deviations were obtained. The groups were compared using the Fisher exact test at a 5% significance level. For urothelial toxicity microscopic examination of urine for haematuria was attempted. Collected urine was centrifuged and the number of red blood cells (RBC) per high-power microscopic field was determined in the sediment (Addis count). Haematuria was graded semiquantitatively as: none = 0–4 RBC, + = 4–10 RBC, + + = 10–20 RBC and + + + = >20 RBC or macrohaematuria. As a further estimation of treatment toxicity survival and body weight change data were used. Toxicity results for both cell lines H 12.1 and 2102 EP were pooled.

**Table I** Characteristics of human testicular cancer cell lines H 12.1 and 2102 EP

| | *H 12.1* | *2102 EP* |
|---|---|---|
| Primary establishment | 21/5/1981 (J. Casper) | 14/10/77 (D. Bronson) |
| Primary histology of the testis tumour | S, EC, CC, DT | TC, YST |
| Histology after heterotransplantation | EC, TC, STGC | EC, STGC |
| Tumour marker production | AFP, β-HCG | AFP (β-HCG) |
| Doubling time *in vivo* | 13.5 days | 9 days |

S, seminoma; EC, embryonal carcinoma; CC, chorioncarcinoma; DT, differentiated teratoma; TC, teratocarcinoma; YST, yolk sac tumour; STGC, syncytiotrophocytic giant cells.

### Results

### *Toxicity of single agents*

The MTD of ifosfamide had been determined in previous experiments to be of the order of 100 mg $kg^{-1}$ $day^{-1}$ (Schmoll, 1989; Harstrick *et al.*, 1990). For mesna given alone concentrations ranging from 5 to 500 mg $kg^{-1}$ $day^{-1}$ were used. No significant toxicity was observed at all concentrations of mesna, resulting in survival of all mice and no apparent anti-tumour activity of this agent. For the experiments mesna at doses between 25 and 100 mg $kg^{-1}$ $day^{-1}$ was used in conjunction with 50 mg $kg^{-1}$ $day^{-1}$ ifosfamide.

### *Toxicity of ifosfamide/mesna combination*

For treatment regimens which used ifosfamide alone or ifosfamide with single-bolus application of mesna at the start of ifosfamide therapy between 37 and 40% of animals had died at day 30. In contrast, schedules using ifosfamide followed by bolus application of mesna at the start of ifosfamide therapy and 3 h later showed a significantly decreased toxicity with only 0–20% of animals dying prior to the end of the experiment (Table II). This tendency could also be observed in the data on body weight change with loss of weight in the range

**Table II** Schedule of ifosfamide/mesna application, toxicity (expressed as number of surving mice and body weight change) and anti-tumour activity calculated as relative tumour area (rTA) in comparison with untreated control mice after 30 days

| | *Toxicity* | | | | *Anti-tumour activity (rTA)* | |
|---|---|---|---|---|---|---|
| | *Survival* | | | *Body weight* | | |
| *Schedule and dosage* | *H 12.1* | *2102 EP* | *Total (%)* | *change (%)* | *H 12.1* | *2102 EP* |
| Control | 5/5 | 5/6 | 94 | −4 ± 2 | 2.40 ± 0.6 | 2.10 ± 0.5 |
| Ifosfamide 50 mg $kg^{-1}$, i.p. on days 1–4 and 15–18 | 3/5 | 4/6 | 63 | −15 ± 5 | 0.80 ± 0.4 | 1.40 ± 0.5 |
| Ifosfamide 50 mg $kg^{-1}$, i.p. Mesna 50 mg $kg^{-1}$, i.p. on days 1–4 and 15–18 | 3/5 | ND | 60 | −10 ± 3 | 0.75 ± 0.2 | ND |
| Ifosfamide 50 mg $kg^{-1}$, i.p. Mesna 25 mg $kg^{-1}$, i.p. at 0 and 3 h on days 1–4 and 15–18 | 5/5 | ND | 100 | −10 ± 2 | 0.68 ± 0.1 | ND |
| Ifosfamide 50 mg $kg^{-1}$, i.p. Mesna 50 mg $kg^{-1}$, i.p. at 0 and 3 h on days 1–4 and 15–18 | 4/5 | 5/6 | 87 | −4 ± 4 | 0.50 ± 0.2 | 1.10 ± 0.3 |

of 10–15% for mice receiving ifosfamide therapy alone compared with 4–10% for animals receiving ifosfamide in combination with two bolus applications of mesna (no statistically significant difference). This trend was observed in both cell lines.

In 16 of 26 mice semiquantitative analysis of urine specimens was possible. Mice receiving ifosfamide in combination with two applications of mesna showed the lowest incidence of haematuria (two of six mice with + haematuria) compared with mice receiving only single-bolus mesna (two of four mice with + haematuria). Of six mice receiving ifosfamide without mesna and examined for erythrocytes in the urine, two mice developed + haematuria, one mouse developed + + haematuria and one mouse macroscopic (+ + +) haematuria.

*Anti-tumour activity of ifosfamide/mesna application*

The anti-tumour activity of different therapy schedules expressed as the relative tumour area (rTA) during the 30 days of the experiment for cell lines H 12.1 and 2102 EP is shown in Figures 1 and 2 respectively. For both cell lines ifosfamide as single-agent therapy showed significant anti-tumour activity with growth retardation of the heterotransplanted tumours. Treatment with ifosfamide alone and treatment with all schedules of ifosfamide plus mesna application resulted in a significant growth reduction of the tumours compared with untreated control mice. In cell line H 12.1 the highest anti-tumour activity was seen with ifosfamide in combination with two bolus applications of mesna at the start of ifosfamide therapy and repeated 3 h later. The application of ifosfamide with mesna at doses from 25 mg $kg^{-1}$ at one time point up to 50 mg $kg^{-1}$ at two different time points did not significantly influence the anti-tumour activity of ifosfamide in comparison with ifosfamide therapy given alone. For cell line 2102 EP both treatment with ifosfamide and ifosfamide plus mesna showed significant activity. No difference in anti-tumour activity was seen between the use of ifosfamide alone compared with ifosfamide with the same dose of mesna given at different time points as bolus administration.

**Figure 1** Growth kinetics of heterotransplanted testicular cancer cell line H 12.1 treated by different schedules of ifosfamide and mesna (rTA = relative tumour area in comparison with untreated controls at day 0). ■, Ifosfamide 50 mg $kg^{-1}$ $day^{-1}$ i.p.; ○, ifosfamide 50 mg $kg^{-1}$ $day^{-1}$ i.p. + mesna 50 mg $kg^{-1}$ $day^{-1}$ i.p.; ●, control; △, ifosfamide 50 mg $kg^{-1}$ $day^{-1}$ i.p. + 2 × mesna 25 mg $kg^{-1}$ $day^{-1}$ i.p. (at 0 and 3 h); ▲, ifosfamide 50 mg $kg^{-1}$ $day^{-1}$ i.p. + 2 × mesna 50 mg $kg^{-1}$ $day^{-1}$ i.p. (at 0 and 3 h) on days 1–4 and 15–18.

**Figure 2** Growth kinetics of heterotransplanted testicular cancer cell line 2102 EP in nude mice after therapy with different schedules of ifosfamide alone and in combination with mesna application. ■, Ifosfamide 50 mg $kg^{-1}$ $day^{-1}$ i.p.; ●, control; ▲, ifosfamide 50 mg $kg^{-1}$ $day^{-1}$ i.p. + 2 × mesna 50 mg $kg^{-1}$ $day^{-1}$ i.p. (at 0 and 3 h) on days 1–4 and 15–18.

### Discussion

Besides cisplatin and etoposide, ifosfamide is one of the most active drugs used in the treatment of testicular cancer. Early phase II studies in the mid-70s have indicated responses in patients with relapsed disease (Schmoll *et al.*, 1978). Since that time similar anti-tumour activity of ifosfamide has been demonstrated in both non-seminomatous and seminomatous germ cell cancer, particularly in combination regimens with cisplatin, bleomycin, vinblastine and etoposide (Schmoll, 1989). Ifosfamide has recently been incorporated into standard regimens for salvage therapy and experimental regimens used for the first-line therapy of patients with advanced disease (Loehrer *et al.*, 1986; Harstrick *et al.*, 1991; Bokemeyer & Schmoll, 1993). With the increasing clinical use in this disease it is a very important result that in a preclinical *in vivo* model with defined experimental conditions the use of mesna at concentrations ranging from 50% to 200% of that of the dose of ifosfamide does not interfere with the anti-tumour activity of ifosfamide, at least in testicular cancer xenografts. Although complete combination crossover dose–response curves of ifosfamide and mesna would have been necessary to definitely exclude any influence of mesna on ifosfamide anti-tumour activity, the data presented here appear sufficiently safe enough to exclude clinically relevant facts. Despite these limitations it may be suitable for influencing different dose intensities and dose escalations within clinical trials.

However, the results gained in the present study are particularly valid for the application of mesna before and after the end of ifosfamide therapy, since this schedule is used clinically because of the long half-life of ifosfamide metabolites in the urine in comparison with a rather short half-life of mesna after intravenous application. The prevention of bladder toxicity of ifosfamide without any impairment of anti-tumour activity by mesna may also be important in view of recent results from phase I/II studies indicating that dose escalation of ifosfamide in patients refractory to standard doses of ifosfamide may be able to achieve clinical responses (Elias *et al.*, 1990).

Different investigators have raised concern about a decreased activity of ifosfamide or cyclophosphamide when given in combination with mesna. Wagner *et al.* (1974)

reported a 50% reduced therapeutic activity of cyclophosphamide in L1210 leukaemia cells heterotransplanted into DBA2 mice when co-administered with mesna. Willemse and de Vries (1989) discussed the problem of reduced ifosfamide activity when combined with a high-dose continuous infusion of mesna compared with single-bolus application mesna in patients with metastatic sarcoma. Two mechanisms have been proposed which might be possibly relevant when continuous high doses (greater than or equal to the dose of ifosfamide) are used:

1. The presence of mesna together with ifosfamide in the blood circulation may inactivate the active drug metabolites 4-hydroxyifosfamide and ifosfamide mustard by the formation of thioethers.
2. *In vitro* studies have shown that the toxic but non-cytostatic ifosfamide metabolite acrolein binds SH groups, thereby reducing the thiol-binding capacity of the cell. This depletion of SH groups may enhance the alkylating potential of other active metabolites with anti-tumour activity. The presence of mesna might reduce this synergistic potentiating effect of acrolein, thereby reducing the cytotoxic activity (Crook *et al.*, 1986; Shaw & Graham, 1987). Others have questioned the quantitative importance of these reactions, but have also proposed to use mesna in the lowest dose that adequately protects the urinary tract (Antman & Elias, 1989).

The use of other substances as selective uroprotective agents has been addressed in different clinical studies. However, many agents investigated, such as carboxycysteine, disulphiram, glutathione, WR 2721 and *N*-acetylcysteine, are mainly cleared from the blood plasma via distribution throughout the tissues and intracellular uptake and the proportion of renal excretion is small. Only a few agents, such as mesna and dimesna, are excreted through the urine (Brock *et al.*, 1984). Two large trials have compared the uroprotective potential of *N*-acetylcysteine versus mesna in patients with soft-tissue sarcomas and germ cell tumours (Munshi *et al.*, 1992; Benjamin *et al.*, 1993). Both studies demonstrated significantly superior results for mesna as a uroprotective agent.

Two additional aspects of mesna application may be important during ifosfamide therapy: in patients undergoing high-dose ifosfamide regimens mesna may not only be able to protect from urothelial toxicity but might also positively influence the incidence of ifosfamide-related central nervous toxicity (Cerny & Küpfer, 1992). Furthermore, the application of mesna may not only prevent acute urothelial damage and haematuria but might also be able to reduce the risk for the occurrence of bladder cancer following cyclophosphamide and ifosfamide therapy (Richie, 1984). *In vitro* results and early clinical trials in patients with superficial bladder cancer have even demonstrated activity of mesna itself as cytotoxic treatment in bladder cancer. Different bladder cancer cell lines were found to be sensitive upon repeated administrations of mesna (Blomgren *et al.*, 1991). The prevention of secondary bladder cancer following oxazophosphorine therapy may be particularly relevant for patients who have a great chance of cure for their primary tumour and who will have a long disease-free life expectancy, such as patients with testicular cancer.

When prophylactic agents are used during chemotherapy for the prevention of specific side-effects caused by the cytotoxic treatment it remains an important issue to prove by the use of adequate tumour models that these prophylactic agents do not interfere with the anti-tumour activity of the cytostatic agent used. Although the number of mice used in the study presented here allows only a limited interpretation of the results without a large statistical analysis, the experiments in two heterotransplanted human testicular cancer cell lines in nude mice show that the application of mesna at clinically used dosages and schedules does not compromise the anti-tumour activity of the oxazaphosphorine derivative ifosfamide. These results may also be relevant for the treatment of other types of cancer with ifosfamide.

The author's would like to thank D. Reile for skilful technical help with the cell culture work.

### References

ANTMAN, K.H. & ELIAS, A. (1989). Mesna: continuous or bolus infusion? (reply to letter). *J. Clin. Oncol.*, **7**, 818.

BENJAMIN, R.S., LEGHA, S.S., PATEL, S.R. & NICAISE, C. (1993). Single-agent ifosfamide studies in sarcomas of soft tissue and bone: the M.D. Anderson experience. *Cancer Chemother. Pharmacol.*, **31** (Suppl. 2), 174–179.

BLOMGREN, H., HALLSTROM, M. & HILLGREN, H. (1991). Antitumor activity of 2-mercaptoethanesulfonate (mesna) *in vitro*. Its potential use in the treatment of superficial bladder cancer. *Anticancer Res.*, **11**, 773–776.

BOKEMEYER, C. & SCHMOLL, H.-J. (1993). Treatment of advanced germ cell tumours by dose intensified chemotherapy with haematopoietic growth factors or peripheral blood stem cells (PBSC). *Eur. Urol.*, **23**, 223–230.

BROCK, N., STEKAR, J., POHL, J., NIEMEYER, U. & SCHEFFLER, G. (1979). Acrolein, the causative factor of urotoxic side-effects of cyclophosphamide, ifosfamide, trofosfamide and sulfosfamide. *Arzneimittelforschung.*, **29**, 659–661.

BROCK, N., HILGARD, P., POHL, J., ORMSTAD, K. & ORRENIUS, S. (1984). Pharmacokinetics and mechanism of action of detoxifying low-molecular-weight thiols. *J. Cancer Res. Clin. Oncol.*, **108**, 87–97.

BRÜHL, P., GÜNTHER, U., HOEFER-JANKER, H., HÜLS, I., SCHEEF, W. & VAHLENSIECK, W. (1976). Results obtained with fractionated ifosfamide massive-dose treatment in generalized malignant tumours. *Int. J. Clin. Pharmacol.*, **14**, 29–39.

BRYANT, B.M., JARMAN, M., FORD, H.T., SMITH, & I.E. (1980). Prevention of isophosphamide-induced urothelial toxicity with 2-mercaptoethane sulphonate sodium (mesnum) in patients with advanced carcinoma. *Lancet*, **ii**, 657–659.

BURKERT, H. (1983). Clinical overview of mesna. *Cancer Treat. Rev.*, **10** (Suppl. A), 175–181.

CABANILLAS, F., HAGEMEISTER, F.B., McLAUGHLIN, P., VELASQUEZ, W.S., RIGGS, S., FULLER, L. & SMITH, T. (1987). Results of MIME-salvage regimen for recurrent or refractory lymphoma. *J. Clin. Oncol.*, **5**, 407–412.

CASPER, J., SCHMOLL, H.-J., SCHNAIDT, U. & FONATSCH, C. (1987). Cell lines of human germinal cancer. *Int. J. Androl.*, **10**, 105–113.

CERNY, T. & KÜPFER, A. (1992). The enigma of ifosfamide encephalopathy. *Ann. Oncol.*, **3**, 679–681.

CROOK, T.R., SOUHAMI, R.L., WHYMAN, G.D. & McLEAN, A.E.M. (1986). Glutathione depletion as determinant of sensitivity of human leukemia cells to cyclophosphamide. *Cancer Res.*, **46**, 5035–5038.

ELIAS, A.D., EDER, J.P., SHEA, T., BEGG, C.B., FREI, E. & ANTMAN, K.H. (1990). High dose ifosfamide with mesna uroprotection. *J. Clin. Oncol.*, **8**, 170–178.

HARSTRICK, A., SCHMOLL, H.-J., CASPER, J., WILKE, H. & POLIWODA, H. (1990). Activity of cytostatic drugs in two heterotransplanted human testicular cancer cell lines with different sensitivity to standard agents. *Eur. J. Cancer.*, **26**, 898–901.

HARSTRICK, A., SCHMOLL, H.-J., WILKE, H., KÖHNE-WÖMPNER, C.H., STAHL, M., SCHÖBER, C., CASPER, J., BRUDEREK, L., SCHMOLL, E., BOKEMEYER, C., BERGMANN, L., LAMMERZ, U., FREUND, M. & POLIWODA, H. (1991). Cisplatin, etoposide and ifosfamide salvage therapy for refractory or relapsed germ cell carcinoma. *J. Clin. Oncol.*, **9**, 1549–1555.

KOVACH, J.S., SCHUTT, A.J., HAHN, R.G., REITEMEIER, R.J. & MOERTEL, C.G. (1974). A phase II study of intermittent high dose isophosphamide therapy of advanced colorectal cancer. *Oncology*, **29**, 34–39.

LOEHRER, P.J., EINHORN, L.H. & WILLIAMS, S.D. (1986). VP-16 plus ifosfamide plus cisplatin as salvage therapy in refractory germ cell cancer. *J. Clin. Oncol.*, **4**, 528–536.

MORGAN, L.R., POSEY, L.E., HITE, S., BICKERS, J.N. & HULL, E.W. (1976). Iphosphamide (Ifosfamide) in the treatment of carcinoma of the lung (abstract). *Clin. Res.*, **24**, 512A.

MORGAN, L.R., POSEY, L.E., HITE, S., BICKERS, J.N. & HULL, E.W. (1981). Ifosfamide: a weekly dose fractionated schedule in bronchogenic carcinoma. *Cancer Treat. Rep.*, **65**, 693–695.

MUNSHI, N.C., LOEHRER, P.J., WILLIMAS, S.D., LANGEFELD, C., SLEDGE, G., NICHOLS, C.R., ROTH, J.B., NEUMAN, A., WALSH, W.B. & EINHORN, L.H. (1992). Comparison of N-acetylcysteine and mesna as uroprotectors with ifosfamide combination chemotherapy in refractory germ cell tumours. *Invest. New Drugs*, **10**, 159–163.

PHILIPS, F.S., STERNBERG, S.S., CRONIN, A.P. & VIDAL, P.M. (1961). Cyclophosphamide and urinary bladder toxicity. *Cancer Res.*, **21**, 1577–1589.

PRATT, C.B., DOUGLASS, E.C., ETCUBANAS, E., GOREN, M.P., GREEN, A.A., HAYES, F.A., HOROWITZ, M.E., MEYER, W.H., THOMPSON, E.I. & WILIMAS, J.A. (1989). Clinical studies of ifosfamide/mesna at St Jude Children's Research Hospital, 1983–1988. *Semin. Oncol.*, **16** (Suppl. 3), 51–55.

RICHIE, K. (1984). Carcinogenicity of antineoplastic agents in men. *Cancer Treat. Rev.*, **11**, 39–67.

SCHMOLL, H.J. (1989). The role of ifosfamide in testicular cancer. *Semin. Oncol.*, **16** (Suppl. 3), 82–93.

SCHMOLL, H.-J., RHOMBERG, W. & DIEHL, V. (1978). Ifosfamide (NSC-109724): Activity in testicular cancer using mono- and combination chemotherapy. In *Current Chemotherapy*, (Vol. 2), Siegenthaler, W. & Luchty, R. (eds), pp. 1089–1091. American Society of Microbiology: Washington.

SHAW, I.C. & GRAHAM, M.I. (1987). Mesna-A (short review). *Cancer Treat. Rev.*, **14**, 67–86.

WAGNER, T., ZINK, M. & SCHWIEDER, G. (1974). Influence of mesna and cysteine on the systemic toxicity and therapeutic efficacy of activated cyclophosphamide. *J. Cancer Res. Clin. Oncol.*, **113**, 160–165.

WANG, J.J., MITTELMANN, A., TWETRINON, P. & SINKS, L.F. (1978). Clinical trial of iphosphamide. *Proc. Am. Assoc. Cancer Res.*, **15**, 110 (abstract 439).

WILLEMSE, P.H.B. & DE VRIES, E.G.E. (1989). Mesna: continuous or bolus infusion? *J. Clin. Oncol.*, **7**, 817–819.

WIST, E.A., SOLHEIM, O.P. & AAMDAL, S. (1987). High-dose concurrent mesna infusion may interfere with the antitumor activity of ifosfamide. In *Ifosfamide in Tumor Therapy (Contributions to Oncology*, Vol. 26), Brade, W., Nagel, G.A. & Seeber, S. (eds), pp. 76–83. S. Karger: Basel.